# Evans syndrome as a presentation in systemic lupus erythematous, coexisting with Hashimoto’s thyroiditis and pernicious anemia: a case report

**DOI:** 10.1186/s13256-024-05002-3

**Published:** 2024-12-28

**Authors:** Maryam Mansour, Arwa Shamasnah, Deema Alsaadi, Saja Abu Saif, Akram krama

**Affiliations:** 1 Faculty of Medicine, Al-Quds University, Jerusalem, Palestine; 2 Istishari Arab Hospital, Ramallah, Palestine

**Keywords:** Evans syndrome, Systemic lupus erythematosus, Autoimmune hemolytic anemia

## Abstract

**Background:**

Evans syndrome is a rare disorder characterized by the simultaneous or sequential combination of autoimmune hemolytic anemia and immunological thrombocytopenia, together with a positive direct antiglobulin test. This syndrome, which can be primary or secondary, is a rare initial manifestation of autoimmune diseases, notably systemic lupus erythematosus, with 1.7–2.7% of patients with systemic lupus erythematosus developing secondary Evans syndrome, sometimes preceding the onset of systemic lupus erythematosus symptoms.

**Case presentation:**

A 47-year-old Middle Eastern female presented with symptoms including shortness of breath, chest pain, and weakness. Physical examination revealed pallor, pale conjunctiva, icteric sclera, tachycardia, and tachypnea. She was diagnosed with Evans syndrome owing to hemolytic anemia, thrombocytopenia, and a positive Coombs test, where initial resistance to treatment prompted intensive therapy with methylprednisolone, intravenous immunoglobulin, and rituximab. Subsequent identification of systemic lupus erythematosus on the basis of positive anti-nuclear antibodies and anti-double-stranded deoxyribonucleic acid antibodies led to treatment with mycophenolate mofetil and hydroxychloroquine. Further evaluations uncovered Hashimoto’s thyroiditis and pernicious anemia, necessitating thyroxine supplementation and vitamin B12 injections.

**Conclusion:**

Hematologic abnormalities play a crucial role in the diagnostic framework of systemic lupus erythematosus. This case highlights a patient initially diagnosed with Evans syndrome, revealing an underlying systemic lupus erythematosus. The presentation underscores the significance of hematologic manifestations as integral components of the diagnostic framework for autoimmune diseases, emphasizing the intricate relationship between Evans syndrome and systemic lupus erythematosus.

## Background

Evans syndrome (ES), initially identified by Evan and Duane in 1951, is an uncommon autoimmune condition of unknown etiology [1], estimated to occur in 1.8 cases per million individuals annually [2]. It is characterized by the concurrent or sequential development of Coombs-positive autoimmune hemolytic anemia (AIHA) and idiopathic thrombocytopenic purpura (ITP), whether or not it is accompanied by immune neutropenia [1]. It is predicted to occur in 7% of patients with AIHA and 2% of ITP cases [3]. It is one of the rare manifestations of autoimmune diseases, particularly systemic lupus erythematosus (SLE), and it occasionally occurs before symptoms ever manifest [4].

The complex interaction among autoimmune disorders is a focal point of interest in the medical field, as recent research uncovers potential connections. This includes the intriguing correlation between SLE and Evans syndrome, and its association with other autoimmune diseases (AIDs) [5–8]. As was predicted, 1.7–2.7% of patients with SLE have been observed to have secondary ES [8, 9]. Additionally, 34% of SLE-associated ES was also linked to other autoimmune diseases, including Takayasu’s arteritis, antiphospholipid syndrome, and even rare autoimmune thyroiditis and autoimmune hepatitis [8]. Very few case reports of the association of SLE, Evans syndrome, Hashimoto’s thyroiditis, and pernicious anemia were found in the literature review, and to the best of our knowledge, our case is one of them.

## Case presentation

We report the case of a 47-year-old white Middle Eastern female, who was apparently in good health with no significant medical history until 1 week prior to admission, when she started to complain of new-onset shortness of breath that increased with exertion and was relieved by rest. This was associated with frequent episodes of central chest pain that increased with activity, generalized weakness, fatigue, and decreased appetite and oral intake. Notably, she described progressive tiredness and observed a yellowish discoloration of her face. Over the course of the week, her symptoms gradually intensified to the point where she experienced dizziness upon standing.

So, she presented to the emergency department and remained hospitalized for 15 days. Initial screening revealed evidence of macrocytic anemia and thrombocytopenia, with a hemoglobin level of 4.5 g/dL, a mean corpuscular volume (MCV) of 120, and a platelet count of 77 × 10^9^/L (Table 1), without signs of active bleeding. Clinical examination revealed an afebrile patient with a pallid appearance, pale conjunctiva, and icteric sclera. Vital signs indicated tachycardia, with a heart rate of 105 beats per minute, and tachypnea, with a respiratory rate of 27 breaths per minute. Other systemic examination yielded normal findings.

**Table 1:** Complete Hematological and Metabolic profile

Result name	Normal range	Results
HCT	37.0–51.0	11.2
Hb	12.0–14.0	4.5
RBC	4.20–6.30	0.93
MCHC	31.0–36.0	40.2
MCH	26.0–32.0	48.4
MCV	80.0–97.0	120.4
MPV	8–11	10.8
PLT	140–440	77
NEUT	1.8–7.70	17.94
LYMPH	1–4.8	4.78
WBC	4–11	23.94
BASO	0.0–0.2	0.02
EO	0.00–0.50	0
PDW	0.1–99.9	11.6
RDW %	11.0–16.0	37.6
CRP	0–5	62.77
Albumin	3.5–5.2	3.88
Creatinine, serum	0.5–0.9	0.52
SGPT (ALT)	10–33	26.6
Blood urea nitrogen	6–20	12.6
SGOT (AST)	0–32	72.4
Chloride	98–107	99.1
Potassium	3.5–5.3	3.09
Sodium	135–145	136
Calcium (Ca), serum	8.4–10	8.51
Mg	1.6–2.6	2
Troponin high sensitivity quantitative	0.008–0.02	0.027

*HCT* Hematocrit; *Hb* Hemoglobin; *RBC* Red blood cell; *MCHC* Mean corpuscular hemoglobin concentration; *MCH* Mean corpuscular hemoglobin; *MPV* Mean platelet volume; *PLT* Platelets; *NEUT* Neutrophils; *LYMPH* Lymhocytes; *WBC* White blood cell; *BASO* Basophils; *EO* Eosinophils; *PDW* Platelet distribution width; *RDW* Red cell distribution width; *CRP* C-reactive protein; *ALT* Alanine aminotransferase; *AST* Aspartate aminotransferase; *Mg* Magnesium

Owing to the patient’s hemodynamic instability, admission to the intensive care unit was warranted. An electrocardiogram (ECG) conducted during this period revealed supraventricular tachycardia (SVT), recurring three times within a 3-day span. The SVT episodes were successfully treated with 6 mg of adenosine. Additionally, two units of least-incompatible packed red blood cell concentrates were transfused. A whole-body computed tomography (CT) scan was performed, which revealed an enlarged spleen that measured 14.5 cm in bipolar length. A peripheral blood smear revealed thrombocytopenia and spherocytes without an increase in the number of schistocytes. Markers for hemolysis (Table 2)were requested, and the results demonstrated a positive direct Coombs test, indicating the presence of autoimmune hemolytic anemia (AIHA).

**Table 2:** Labratory results for Hemolytic workup

Test name	Normal range	Results
Bilirubin (direct)	0–0.2	0.748
Bilirubin (total)	0–1.2	2.180
Lactate dehydrogenase total (LDH)	135–214	836
Haptoglobin	0.3–2	0.01
Reticulocytes %		25.84
Direct Coombs test (DCT)		Positive + 4 (IgG) / + 2 (C3d)
Indirect Coombs test (ICT)		Positive + 3

Viral serologies (Table 3), along with blood, nasal, and rectal swabs, were collected, and cultures were negative, ruling out a current infection. Furthermore, a transthoracic echocardiogram was conducted, yielding normal findings. Further investigation through bone marrow aspiration and biopsy revealed a cellularly reactive bone marrow with erythroid hyperplasia and no morphologic evidence of infiltrative disease or leukemia.

**Table 3:** Viral and Serological testing results

Test name	First day of admission	Fifth day of admission	Seventh day of admission
HIV Abs screening	Nonreactive		
Epstein–Barr virus IgG/IgM	Reactive/NR		
HCV	Nonreactive		
HBsAg screening	Nonreactive		Nonreactive
HBc total		Reactive	
HBc IgM		Nonreactive	
HBsAb (anti-s)			Reactive 87.3
HBe Ab			Reactive

*HIV* Human immunodeficiency virus; *HCV* Hepatitis C virus; *HBsAg* Hepatitis B surface antigen; *HBc total* Hepatitis B core antibody total; *HBsAb* Hepatitis B surface antibody; *HBeAb* Hepatitis B Envelop antibody

In light of the hemolytic anemia, thrombocytopenia, and positive direct antiglobulin test (DAT) and after ruling out other possible diagnoses, including thrombotic thrombocytopenic purpura, disseminated intravascular coagulation, paroxysmal nocturnal hemoglobinuria, hemolytic-uremic syndrome, antiphospholipid syndrome, and drug-induced AIHA, a clinical diagnosis of Evans syndrome was established.

Upon confirming the diagnosis of Evans syndrome, the patient was initiated on an intensive therapeutic regimen after showing resistance to the initial treatment with corticosteroids. This began with a pulse therapy of methylprednisolone at 1 g × 1 for 3 days. Simultaneously, a course of 40 g of IVIG 2 g/kg for 4 days was initiated. In addition, four doses of rituximab at a dosage of 375 mg/m^2^ weekly for 4 weeks were administered. The first two doses were administered while she was in hospital, while the other two doses were administered weekly as an outpatient. After this comprehensive intervention, clinically, the patient became asymptomatic, and her CBC and hemolytic lab tests, including reticulocytes count and LDH, returned to normal.

Throughout the patient’s hospitalization, the emergence of a malar rash on the face, and the occurrence of oral ulceration, prompted the requisition of rheumatological markers. The results revealed positive anti-nuclear antibodies (ANA), positive anti-DsDNA, a decreased C4 level at 4 mg/dL (with a normal range of 10–40 mg/dL), and an elevated ESR level of 150 (compared with the normal range of less than 20 mm/hour). Notably, the extractable nuclear antigen (ENA) profile yielded negative findings except for SS-A native (SS A/RO60). This constellation of clinical and laboratory evidence aligns with the diagnostic criteria for systemic lupus erythematosus (SLE), as stipulated by the American College of Rheumatology (ACR) [10]. Consequently, a diagnosis of Evans syndrome concomitant with SLE was conclusively established.

Following this, mycophenolate mofetil was introduced at 500 mg twice daily. Considering the patient’s positive hepatitis B virus core status, lamivudine was initiated at a dosage of 100 mg once daily. Hydroxychloroquine was introduced at 200 mg twice daily as part of the ongoing therapeutic strategy. After this comprehensive management, the patient was discharged and commenced rheumatologic disease outpatient follow-up.

A few months later, during the patient’s ongoing evaluation, the patient reported fatigue and significant weakness. Additional laboratory tests and an autoimmune panel were conducted to explore the possibility of other autoimmune diseases. The results revealed an elevated TSH level at 5.85 mU/L (normal range 0.35–4.94 mU/L), a reduced free T4 level at 0.5 mU/L (normal range 0.7–1.48 mU/L), and positive findings for anti-thyroglobulin antibody and anti-thyroid peroxidase antibody, which confirmed the diagnosis of Hashimoto’s thyroiditis. Furthermore, the tests indicated deficient vitamin B12 levels, and the confirmation of anti-parietal cell and anti-intrinsic factor antibodies confirmed the diagnosis of pernicious anemia. In response to these findings, the patient initiated a therapeutic regimen consisting of thyroxine at 25 mcg and intramuscular vitamin B12 injections. A year later, her general symptoms, including those associated with SLE, as well as her laboratory values, including CBC and liver function, had returned to normal.

## Discussion

We present a case of systemic lupus erythematosus (SLE) manifesting as Evans syndrome. Our patient presented with shortness of breath, chest pain, fatigue, jaundice, and orthostatic hypotension. Evans syndrome was confirmed as the initial diagnosis, characterized by a combination of AIHA and ITP. Subsequently, SLE was identified on the basis of positive Ds-DNA and positive ANA. Patients diagnosed with SLE should be considered for other autoimmune disorders. Considering the association of SLE with other autoimmune conditions, a comprehensive autoimmune panel was conducted. The results unveiled the coexistence of Hashimoto’s thyroiditis and pernicious anemia, highlighting the significance of thorough evaluation in patients diagnosed with SLE.

Evans syndrome is a rare disorder, and the most documented cases are in children [11–14]. Consequently, there is limited information available regarding the symptoms and management of Evans syndrome in adults. The disorder is categorized as primary or secondary, and it has links to a number of conditions, including infections, lymphoproliferative disorders, primary immunodeficiency, and autoimmune disorders including rheumatoid arthritis and systemic lupus erythematosus (SLE) [2]. Approximately 3–15% of individuals with Evans syndrome eventually develop SLE [15]. Patients with lupus frequently have hematologic symptoms, such as anemia, thrombocytopenia, pancytopenia, and leukopenia [16]. In our case, secondary Evans syndrome was the initial presentation of SLE, which is a rare manifestation of the disease.

While diagnosing Evans syndrome (ES), it is crucial to distinguish between primary and secondary cases, as this can impact the treatment approach [5]. The first-line treatment typically involves corticosteroids, which may induce remission, but they can also lead to exacerbations and recurrences. For refractory cases, second-line treatment options include intravenous immunoglobulins (IVIG), rituximab, splenectomy, cyclosporine, or azathioprine [5, 17]. The prompt and accurate recognition of Evans syndrome facilitated the identification of appropriate therapy involving immunosuppressive agents, contributing to a rapid recovery and the restoration of hematologic function [5, 8, 17, 18].

Despite therapy, the vast majority of patients with ES experience persistent relapses, which are linked to a high risk of morbidity and mortality [5, 17]. So, its management is still a challenge. Furthermore, it should be noted that the majority of the suggestions made about treatment are derived from single ITP or isolated AIHA cases, as there are very few clinical studies comparing different treatment modalities because of the disease’s rarity [3].

The goal of presenting this case is to emphasize the need for awareness of this rare entity. A high index of suspicion among primary care physicians and other specialties is crucial. If observed, it is essential to conduct screening for secondary causes, as their early detection enables timely and suitable intervention.

## Conclusion

Systemic lupus erythematosus (SLE) is a multi-system autoimmune disease that affects any organ in the body. In rare instances, the initial presentation of SLE may manifest as Evans syndrome, which is also an autoimmune disease that includes two or three cytopenias presenting as autoimmune hemolytic anemia and immune thrombocytopenia with or without immune neutropenia. Additionally, other autoimmune diseases may coexist in the same patient. As these diseases are all about immunity, the treatment certainly will be centralized on suppression of the immune system using immunosuppressant drugs, with an emphasis on the idea that the treatment of SLE differs from the treatment of both diseases presenting at the same time in the same patient. However, treatment in the presence of Evans syndrome with SLE is more challenging because of the risk of many relapses and increasing morbidity and mortality.

## Data Availability

Information related to the case is available from the corresponding author upon reasonable request.

## Abbreviations

SLE: Systemic lupus erythematosus
ES: Evans syndrome
AIHA: Autoimmune hemolytic anemia
ITP: Idiopathic thrombocytopenic purpura
AIDs: Autoimmune diseases
MCV: Mean corpuscular volume
ECG: Electrocardiogram
SVT: Supraventricular tachycardia
CT: Computed tomography
DAT: Direct antiglobulin test
IVIG: Intravenous immunoglobulin
ANA: Anti-nuclear antibodies
ESR: Erythrocyte sedimentation rate
Ds-DNA: Anti-double-stranded deoxyribonucleic acid antibodies
ENA: Extractable nuclear antigen
ACR: American College of Rheumatology
TSH: Thyroid stimulating hormone
